# A combination of risk stratification systems for thyroid nodules and cervical lymph nodes may improve the diagnosis and management of thyroid nodules

**DOI:** 10.3389/fonc.2024.1393414

**Published:** 2024-06-27

**Authors:** Cong-Ying Xu, Jing Yu, Yi-Yang Cui, Yuan-Jing Huang, Chao Fu, Ke-Fei Cui

**Affiliations:** Department of Ultrasound, The First Affiliated Hospital of Zhengzhou University, Zhengzhou, China

**Keywords:** thyroid nodules, lymph nodes, risk stratification system, management, ultrasound

## Abstract

**Introduction:**

To assess the performance of the European Thyroid Association Thyroid Imaging and Reporting Data System (EU-TIRADS) and the Korean Thyroid Imaging Reporting and Data System (K-TIRADS), which combine risk stratification systems for thyroid nodules (TN-RSS) and cervical lymph nodes (LN-RSS) in diagnosing malignant and metastatic thyroid cancer in a single referral center.

**Methods:**

We retrospectively analyzed 2,055 consecutive patients who underwent thyroidectomy or fine-needle aspiration (FNA) from January 2021 to December 2022. TNs and LNs were categorized according to the ultrasonography (US) features of EU-TIRADS and K-TIRADS, respectively. The diagnostic performance and postponed malignancy rate (PMR) were compared with those of EU-TIRADS and K-TIRADS. PMR was defined as the number of patients with malignant nodules not recommended for biopsy among patients with cervical LN metastasis.

**Results:**

According to the EU-TIRADS and K-TIRADS, for TN-RSS alone, there were no significant differences in sensitivity, specificity, accuracy, unnecessary FNA rate (UFR), missed malignancy rate (MMR), and PMR between the two TIRADSs (29.0% vs. 28.8%, 50.5% vs. 51.1%, 32.3% vs. 32.2%, 23.6% vs. 23.5%, 88.6% vs. 88.5%, and 54.2% vs. 54.5%, P > 0.05 for all). Combining the LN-RSS increased the diagnostic accuracy (42.7% vs. 32.3% in EU-TIRADS; 38.8% vs. 32.2% in K-TIRADS) and decreased the PMR (54.2% vs. 33.9% in EU-TIRADS; 54.5% vs. 39.3% in K-TIRADS). EU-TIRADS had higher sensitivity and accuracy and lower PMR than K-TIRADS (41.3% vs. 36.7%, 42.7% vs. 38.8%,33.9% vs. 39.3%, P < 0.05 for all).

**Conclusions:**

A combination of TN-RSS and LN-RSS for the management of thyroid nodules may be associated with a reduction in PMR, with enhanced sensitivity and accuracy for thyroid cancers in EU-TIRADS and K-TIRADS. These results may offer a new direction for the detection of aggressive thyroid cancers.

## 1 Introduction

With the wide application of US and FNA technology in the diagnosis of thyroid nodules, the incidence of thyroid cancer has increased significantly worldwide (1). The LN metastasis rate of differentiated thyroid carcinomas (including papillary thyroid carcinoma) is as high as 60%–70% (2, 3). For patients with differentiated thyroid carcinomas, precise identification of LN metastases is crucial. Assessments of the situation of LN metastasis affect the surgical modalities and prognosis of patients (4–8). Therefore, some guidelines also suggest the necessity of managing thyroid nodules in conjunction with LNs. The condition of cervical LNs is also the focus of thyroid examination; however, only a few international guidelines propose concrete management strategies for cervical LNs.

Recently, the EU-TIRADS proposed by the European Thyroid Association and the K-TIRADS proposed by the Korean Society of Thyroid Radiology/Korean Thyroid Association have raised lymph node risk classifications (LN-RSS) similar to those of thyroid nodules (TN-RSS) (9–11). The K-TIRADS and EU-TIRADS comprise definitions of benign and low-, intermediate-, and high-risk nodules and indications for FNA. They also classify LNs into three categories, including the K-TIRADS (probably benign, indeterminate, and suspicious categories) and the EU-TIRADS (normal, indeterminate, and suspicious for malignancy categories), and propose recommended FNA indications. LN metastasis is a crucial indicator because it increases the extent of surgical resection and elevate persistent or recurrent disease and repeat surgery complications for patients with cervical LN metastasis who have been underdiagnosed or misdiagnosed (12). The failure of the examination to reveal metastatic LNs will result in a postponed diagnosis for the patient. Unfortunately, there are currently no studies evaluating postponed malignancy rate (PMR).

Therefore, we hypothesize that a combination of TN-RSS and LN-RSS for the management of thyroid nodules might be an effective strategy per EU-TIRADS and K-TIRAD, leading to a decrease in PMR.

## 2 Methods

This study involving human participants was reviewed and approved by the scientific research and clinical trials ethics committee of The First Affiliated Hospital of Zhengzhou University of China. Written informed consent for the use of data was waived.

### 2.1 Patients

**Figure 1:**
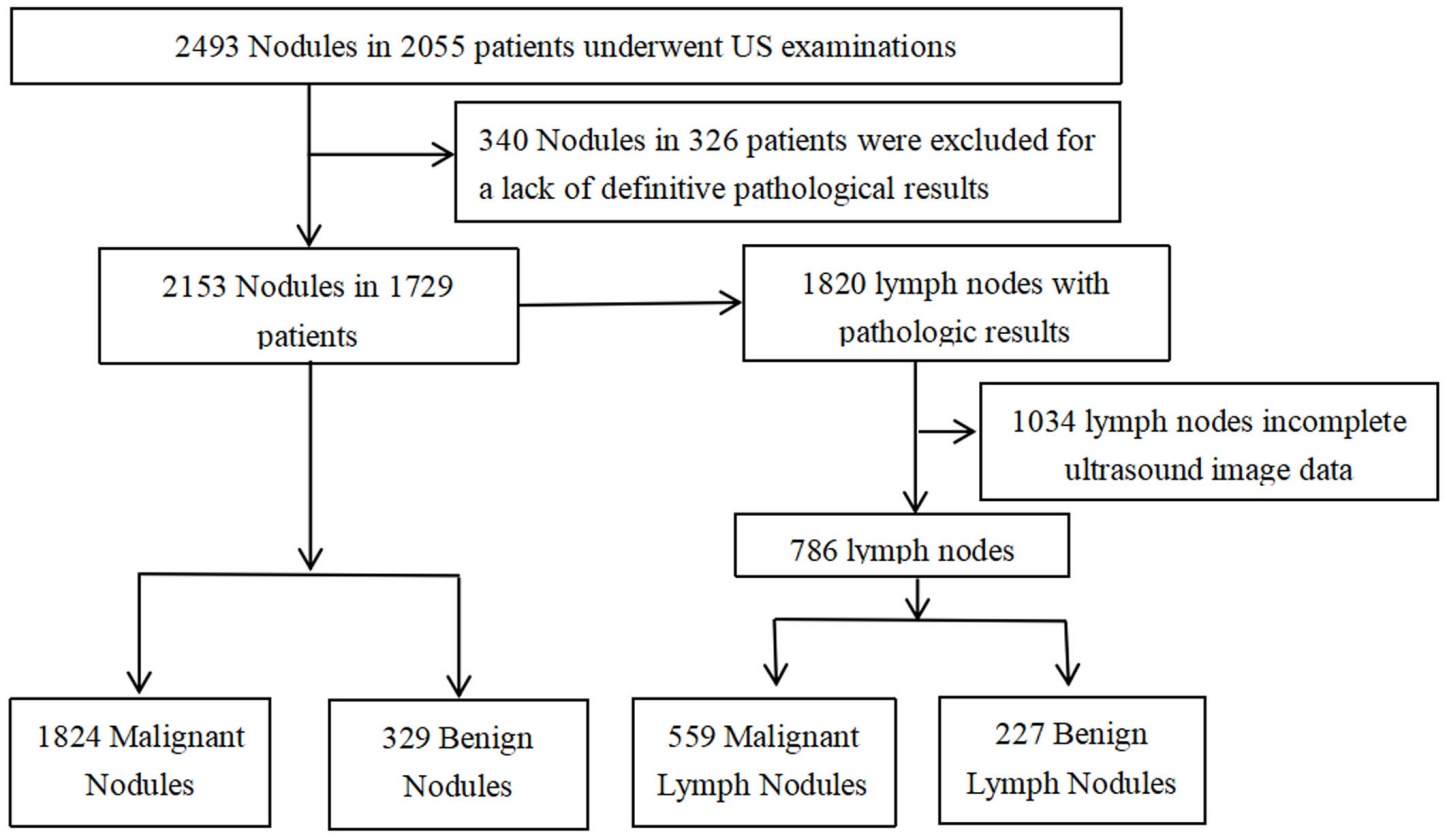
Flowchart showing the recruitment of study participants.

From January 2021 to December 2022, 2,493 thyroid nodules in 2,055 consecutive patients were examined by ultrasonography (US) and confirmed pathologically by surgery or puncture. The inclusion criteria were as follows (1): US was performed preoperatively and (2) surgical or FNA pathology performed within one month of US examinations. The exclusion criteria were as follows (1): incomplete ultrasound image data and (2) lack of definitive pathological findings (Less than 1 year follow-up for pathologically benign). Ultimately, 2,153 thyroid nodules from 1,729 patients were included in this study. With 1,820 LNs with pathological findings associated with the included 2,153 nodes, only 786 LNs ultimately remained available for this study (**Figure 1**).

### 2.2 Ultrasound examination and imaging analysis

All US examinations were performed using a 5–14-MHz linear probe and a real-time US system (Toshiba Aplio300 or IU22), and they were all performed by senior radiologists with more than 6 years of experience in thyroid imaging. A senior radiologist with 9 years of clinical experience in thyroid US scanning and thyroid US image evaluation observed and recorded the following findings: i) Thyroid nodules: size, location, composition (solid or almost solid, predominantly solid, predominantly cystic, cystic, and spongiform), echogenicity (hyperechoic, isoechoic, hypoechoic, and marked hypoechoic), shape (parallel, nonparallel), margins (smooth, lobulated, irregular, and ill-defined), condition of trachea invasion, number (single or multiple), condition of capsular involvement, condition of calcification echogenic foci (punctate echogenic foci, macrocalcification, peripheral calcifications, and comet-tail artifacts); ii) The shape, structure, and blood flow distribution of cervical LNs were observed to determine whether they were normal or not. The cervical LNs were recorded as follows: size (the long and short diameters were both measured in the most representative longitudinal nodal plane showing the LN maximum and minimum diameters), location (levels I–VI), L/S ratio, lymphatic hilum, punctate echogenic foci (echogenic foci ≤ 1 mm), large echogenic foci (>1 mm) in the LNs, liquefied or cystic appearance in the LN, cortical hyperechogenicity in the LN, and abnormal vascularity (peripheral or diffuse).

### 2.3 Classification of thyroid nodules and cervical lymph nodes

For nodules, the 2021 K-TIRADS states that a nodule is significantly associated with malignancy when a solid hypoechoic nodule is accompanied by any of the three suspicious US features (punctate echogenic foci, nonparallel orientations, and irregular margins); similarly, the 2017 EU-TIRADS states that a suspected malignant nodule manifests itself as at least one of the following highly suspicious features (nonoval shape, irregular margins, microcalcifications, marked hypoechogenicity (and solid)). Biopsy size thresholds for nodules: K-TIRADS 3 and EU-TIRADS 3 > 2.0 cm, K-TIRADS 5 and EU-TIRADS 5 > 1.0 cm, and K-TIRADS 4 and EU-TIRADS 4 > 1.5 cm for this study (K-TIRADS 4 > 1.0–1.5 cm in the original version).

For LNs, LNs are classified into three categories (probably benign, indeterminate, and suspicious) by K-TIRADS, and cervical LNs are recommended for FNA indications (indeterminate LNs: size >5 mm, suspicious LNs: size >3–5 mm); EU-TIRADS LN classifications (normal, indeterminate, and suspicious for malignancy); and US-FNA will be recommended for LNs (suspicious and indeterminate LNs). Unfortunately, LNs cannot be included by EU-TIRADS when LNs are present as normal hilum, oval shape, normal size; normal hilum and increased size (short axis ≥8 mm in level II and ≥5 mm in level III or IV); normal hilum and round shape. They are all classified together as “unspecified” LNs. Meanwhile, “unspecified” LNs with an increased short axis (short diameter ≥ 5 mm) were still chosen to undergo FNA in this study.

Finally, two reviewers (with 9 years and 12 years of clinical experience in thyroid US image evaluation), with confidentiality of biopsy results and final pathological diagnosis, classified thyroid nodules and LNs based on US features. They conferred and made the final decision when classifications did not agree. It was also determined whether each nodule and LN reached the thresholds based on size, TN-RSS, and LN-RSS. FNA was performed when thyroid nodes reached their thresholds in EU-TIRADS and K-TIRADS, and both LNs and the corresponding thyroid nodes performed FNA when LNs reached the thresholds.

### 2.4 Analysis and comparison of diagnostic performance

In this study, we investigated the diagnostic performance of calculating and comparing the diagnostic performance of the simple criteria (TN-RSS) and the dual criteria (TN-RSS and LN-RSS) based on the final assessment categories and size thresholds in EU-TIRADS and K-TIRADS. Diagnostic performance was estimated by calculating the sensitivity, specificity, accuracy, unnecessary FNA rate (UFR; number of benign nodules recommended for biopsy), missed malignancy rate (MMR; number of malignant nodules among nodules not recommended for biopsy), and PMR (number of patients with malignant nodules not recommended for biopsy among patients with cervical LN metastases).

### 2.5 Statistical analysis

Demographic features were compared between participants with benign and malignant nodules using the two-sample t-test for continuous data and the chi-square test for categorical data. The malignancy rates according to TN-RSS and the combination of TN-RSS and LN-RSS in the two TIRADSs were calculated as percentages. The diagnostic performances and UFR, MMR, and PMR (along with their 95% confidence intervals) were calculated and compared using the McNemar test or Pearson test. Statistical analyses were performed using SPSS 26.0 and MedCalc 20.0.22 software. The difference was considered statistically significant at a two-sided P < 0.05.

## 3 Results

### 3.1 Pathological diagnosis

Of the 2,153 thyroid nodules, 1,824 (84.7%) were diagnosed as malignant and 329 (15.3%) as benign by pathology. Papillary thyroid carcinomas were found to be the most common malignant nodules (1,794 papillary thyroid carcinomas, 10 follicular carcinomas, 11 medullary carcinomas, and 9 others). Adenomatous hyperplasia was the most common benign nodule (291 adenomatous hyperplasia, 20 follicular adenomas, 3 nodular hyperplasia, 10 inflammatory lesions, and 5 others). Of the studied LNs, 214 were subjected to preoperative FNA and washout thyroglobulin (Tg) measurement, of which 186 and 28 were malignant (all surgically resected) and benign (no significant change at 1-year follow-up). Eventually, 786 were included: 559 (71.1%) were metastatic and 227 (28.9%) were benign.

### 3.2 Baseline clinicopathological characteristics

The demographics and US features of the patients and nodules are summarized in **Table 1**. Patients aged less than 55 years had a higher malignancy rate than those aged ≥55 years (P < 0.05). Benign nodules were larger than malignant nodules (22.0 ± 17.5 mm vs. 9.9 ± 8.2 mm, P < 0.001). Male patients had a significantly higher rate of malignancy of nodules than female patients (88.7% vs. 83.5%, P < 0.05). There were 1,360 (63.2%) nodules measuring <10 mm and 793 (36.8%) nodules measuring ≥10 mm in diameter. The malignancy rate in nodules with a diameter of <10 mm was higher than that in nodules with diameters of ≥10 mm (67.9% vs. 32.1%, P < 0.05).Almost all malignant nodules were solid or almost solid (97.4%). Compared with benign nodules, malignant nodules were more hypoechoic or marked hypoechoic (99.1% vs. 66.3%), their margins were more often irregular or lobulated (49.3% vs. 8.9%), and they showed more punctate echogenic foci (59.4% vs. 19.1%). All suspicious features documented on thyroid ultrasound were significantly more frequent in malignant nodules than in benign nodules.

**Table 1:** Summary of demographic and US features for the patients with thyroid nodules.

Characteristics	Final pathology		Total	p
	Benign	Malignant		
No. Of nodules	329	1,824	2,153	
Age				0.000
Mean (years)	48.9 ± 12.7	44.2 ± 11.5	44.9 ± 11.8	
Range (years)	14–77	9–79	9–79	
<55	211 (64.1)	1,459 (80.0)	1,670 (77.6)	0.000
≥55	118 (35.9)	365 (20.0)	483 (22.4)	
Gender				0.005
Male	56 (17.0)	440 (24.1)	496 (23.0)	
Female	273 (83.0)	1,384 (75.9)	1,657 (77.0)	
Size				0.000
Mean (mm)	22.0 ± 17.5	9.9 ± 8.2	11.7 ± 11.1	
Range (mm)	2.5–70.0	1.8–81.0	1.8–81.0	
<10mm	122 (37.1)	1,238 (67.9)	1,360 (63.2)	0.000
≥10mm	207 (62.9)	586 (32.1)	793 (36.8)	
Ultrasound Features				
Composition				0.000
Mixed cystic and solid	116 (35.3)	47 (2.6)	163 (7.6)	
Solid or almost solid	213 (64.7)	1,777 (97.4)	1,990 (92.4)	
Echogenicity				0.000
Isoechogenicity	101 (30.7)	17 (0.9)	118 (5.5)	
Hyperechogenicity	10 (3.0)	0 (0.0)	10 (0.5)	
Hypoechogenicity	202 (61.4)	1,260 (69.1)	1,462 (67.9)	
Marked hypoechogenicity	16 (4.9)	547 (30.0)	563 (26.1)	
Shape				0.000
Parallel	256 (77.8)	585 (32.1)	841 (39.1)	
Nonparallel	73 (22.2)	1,239 (67.9)	1,312 (60.9)	
Margin				0.000
Smooth	163 (49.5)	109 (6.0)	272 (12.6)	
Ill-defined	137 (41.6)	816 (44.7)	953 (44.3)	
Irregular or lobulated	29 (8.9)	899 (49.3)	928 (43.1)	
Echogenic foci				0.000
No	193 (58.7)	660 (36.2)	853 (39.6)	
Comet-tail	22 (6.7)	6 (0.3)	28 (1.3)	
Macrocalcifications	45 (13.7)	72 (3.9)	117 (5.4)	
Peripheral calcifications	6 (1.8)	4 (0.2)	10 (0.5)	
Punctate echogenic foci	63 (19.1)	1,082 (59.4)	1,145 (53.2)	

Data in parentheses are percentages.

### 3.3 Comparison of diagnostic performance according to the combination of TN-RSS and LN-RSS in EU-TIRADS and K-TIRADS

**Figure 2:**
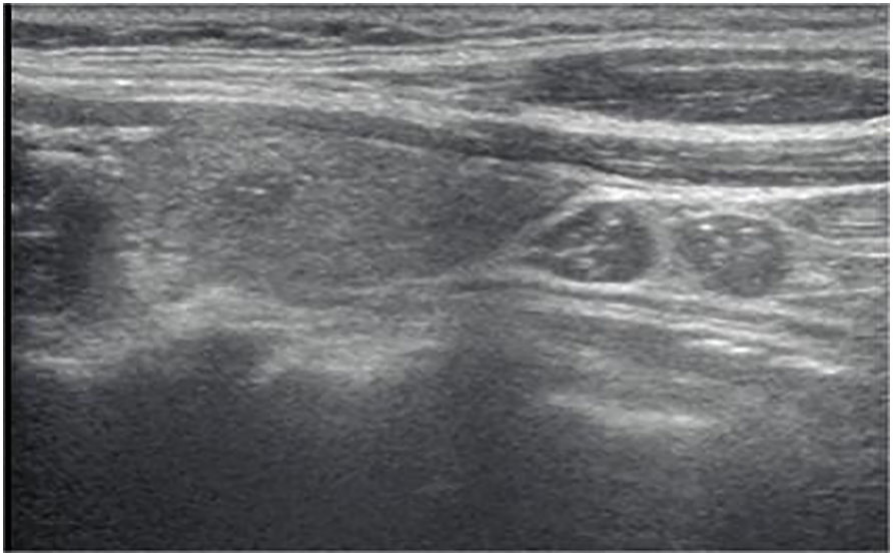
Longitudinal ultrasonography image from a 39-year-old woman with papillary thyroid carcinoma shows a 6-mm solid, hypoechoic, and ill-defined margin thyroid nodule with punctate echogenic foci, associated with LN metastases presenting as diffuse hyperechogenicity with punctate echogenic foci, L/S<2.The nodule was classified as EU-TIRADS 5:high risk or K-TIRADS 5:high suspicion. The LNs in level VI were classified as “suspicious for malignancy” according to the EU-TIRADS category and “suspicious” for the K-TIRADS category. The FNA failed based on nodule size, while according to the LNs was suggested.

**Figure 3:**
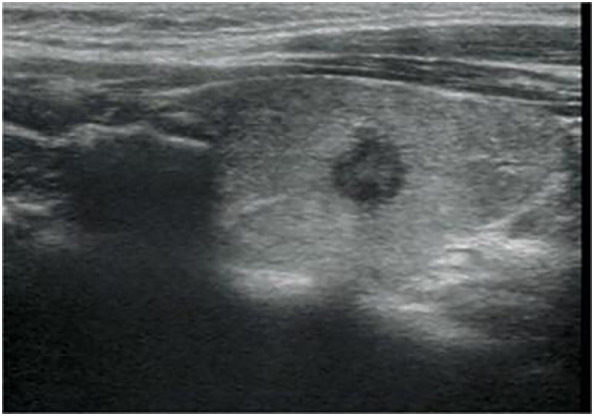
Longitudinal ultrasonography image from a 34-year-old woman with papillary thyroid carcinoma shows a 7.5-mm solid, hypoechoic, and irregular margin thyroid nodule with punctate echogenic foci, associated with no suspicious lymph node. The nodule was classified as EU-TIRADS 5:high risk or K-TIRADS 5:high suspicion. The nodular failed FNA based on nodule size.

EU-TIRADS and K-TIRADS (the combination of TN-RSS and LN-RSS) improved sensitivity and accuracy and reduced the PMR (**Table 2**, **Figures 2** and **3**). However, EU-TIRADS had significantly higher sensitivity and accuracy and a significantly lower PMR than K-TIRADS (41.3% vs. 36.7%, 42.7% vs. 38.8%,33.9% vs. 39.3%, P < 0.05 for all, **Table 3**).

**Table 2:** Comparison of the diagnostic performances between TN-RSS alone and the combination of TN-RSS and LN-RSS in the EU-TIRADS and K-TIRADS.

	Sensitivity	Specificity	Accuracy	UFR	MMR	PMR
EU-TN	29.0%	50.5%	32.3%	23.6%	88.6%	54.2%
EU-TN + LN	41.4%	49.9%	42.7%	17.9%	86.7%	33.9%
P-value	0.000	0.500	0.000	0.006	0.126	0.000
K-TN	28.8%	51.1%	32.2%	23.5%	88.5%	54.5%
K-TN + LN	36.7%	50.8%	38.8%	19.5%	87.4%	39.3%
P-value	0.000	1.000	0.000	0.060	0.338	0.000

TN-RSS, the risk stratification system for thyroid nodules; LN-RSS, the risk stratification system for lymph nodes; TIRADS, Thyroid Imaging Reporting and Data System; UFR, unnecessary FNA rate; MMR, missed malignancy rate; PMR, postponed malignancy rate.

**Table 3:** Diagnostic performances of the combination of TN-RSS and LN-RSS in EU-TIRADS and K-TIRADS.

	Sensitivity	Specificity	Accuracy	UFR	MMR	PMR
EU-TN + LN	41.4%	49.9%	42.7%	17.9%	86.7%	33.9%
K-TN + LN	36.7%	50.8%	38.8%	19.5%	87.4%	39.3%
P-value	0.000	0.375	0.000	0.403	0.615	0.000

TN-RSS, the risk stratification system for thyroid nodules; LN-RSS, the risk stratification system for lymph nodes; TIRADS, Thyroid Imaging Reporting and Data System; UFR, unnecessary FNA rate; MMR, missed malignancy rate; PMR, postponed malignancy rate.

### 3.4 Comparison of diagnostic performance according to TN-RSS alone in EU-TIRADS and K-TIRADS

Diagnostic performances were calculated according to FNA thresholds and compared between EU-TIRADS and K-TIRADS (**Table 4**). There were no significant differences in sensitivity, specificity, accuracy, UFR, MMR, and PMR between the two TIRADSs (29.0% vs. 28.8%, 50.5% vs. 51.1%, 32.3% vs. 32.2%, 23.6% vs. 23.5%, 88.6% vs. 88.5%, and 54.2% vs. 54.5%, P > 0.05 for all).

**Table 4:** Diagnostic performances of TN-RSS alone in the EU-TIRADS and K-TIRADS.

	Sensitivity	Specificity	Accuracy	UFR	MMR	PMR
EU-TN	29.0%	50.5%	32.3%	23.6%	88.6%	54.2%
K-TN	28.8%	51.1%	32.2%	23.5%	88.5%	54.5%
P-value	0.289	0.626	0.774	0.970	0.939	0.500

TN-RSS, the risk stratification system for thyroid nodules; TIRADS, Thyroid Imaging Reporting and Data System; UFR, unnecessary FNA rate; MMR, missed malignancy rate; PMR, postponed malignancy rate.

### 3.5 US features of the central and lateral regions for diagnosing metastatic LNs from thyroid cancer and LN classifications based on EU-TIRADS and K-TIRADS

For metastatic LNs, the LNs in the lateral region were more likely to exhibit size enlargement, cystic changes, punctate echogenic foci, cortical hyperechogenecity, and abnormal vascularity, whereas those in the central region were more likely to exhibit L/S < 2 with absent hilum (P < 0.05 for all, **Table 5**). In the assessment of LN-RSS, 150 of 786 LNs (19.1%) did not meet the criteria for any pattern and were thus classified as “not specified.” The malignancy risk of LNs classified as “not specified” was 36.7% (55/150 LNs).

**Table 5:** Ultrasonography features of metastatic lymph nodes of lateral and central neck.

Characteristics	lateral	central	P
Short diameter (mm)	15.9 ± 8.4	8.1 ± 3.5	0.000
Long diameter (mm)	7.7 ± 4.3	4.5 ± 2.1	0.000
L/s ratio < 2	104 (45.6)	218 (65.9)	0.000
L/s ratio ≥ 2	124 (54.4)	113 (34.1)	
Hilum			0.363
Present	5 (2.2)	4 (1.2)	
Absent	223 (97.8)	327 (98.8)	
Cystic changes	76 (33.3)	13 (3.9)	0.000
Punctate echogenic foci	191 (83.8)	135 (40.8)	0.000
Abnormal vascularity	167 (73.2)	81 (24.5)	0.000
Cortical hyperechogenecity	110 (48.2)	21 (6.3)	0.000

Data are expressed as the number of cases with percentages in parentheses unless otherwise indicated. *Data are expressed as mean ± standard deviation. L/S ratio = long-to-short diameter ratio.

The malignancy rate at each level is shown in **Table 6**. The malignancy rates for the normal, indeterminate, suspicious, and “not specified” categories in EU-TIRADS were 0%, 50.5%, 90.3%, and 36.7%, The malignancy rate of the modified indeterminate category in EU-TIRADS was 44.4% (151/340) when “not specified” was categorized as the intermediate category. Those for the probably benign, indeterminate, and suspicious categories of K-TIRADS were 16.7%, 44.2%, and 90.3%.

**Table 6:** Malignancy rate of lymph nodes classified according to the EU-TIRADS and K-TIRADS.

	Number of Cases	Metastatic Lymph Nodes	Malignancy Rate (%)
EU-TIRADS category			
Normal	5	0	0.0
Indeterminate	190	96	50.5
Suspicious for malignancy	289	261	90.3
Not specified	150	55	36.7
K-TIRADS category			
Probably benign	6	1	16.7
Indeterminate	339	150	44.2
Suspicious	289	261	90.3

TIRADS, Thyroid Imaging Reporting and Data System.

## 4 Discussion

Herein, K-TIRADS and EU-TIRADS were evaluated based on the nodules criteria, and there was no significant difference in diagnostic efficacy between the two, similar to the results of the previous study. Considering that tumor size is closely related to prognosis, TN-RSS recommends a combination of size cutoffs and US features to provide a rationale for FNA (9, 11, 13–15). Unfortunately, TN-RSS alone may lead to individuals with cervical LN metastases not having a thyroid nodule programmed for biopsy (16). Based on the findings of our study, a combination of TN-RSS and LN-RSS for the management of thyroid nodules may be associated with a reduction in PMR while improving sensitivity and accuracy, according to EU-TIRADS and K-TIRADS. Furthermore, EU-TIRADS seems to be performing better than K-TIRADS, despite the loss of some ease of operation.

On the one hand, many studies have suggested that a higher threshold for FNA contributes to the reduction of unnecessary biopsy recommendations and the prevention of overdiagnosis and overtreatment (17–21). Conversely, thyroid carcinoma with underdiagnosed or misdiagnosed metastatic LNs was postponed, causing irreversible negative consequences (5, 6). LNs with ≤2 mm micrometastases in the central neck region are not easily visualized and are not associated with a risk of disease recurrence (6, 22, 23); however, ultrasound-visible >3 mm LNs identified preoperatively are associated with detection and removal (24). Thyroid nodules measuring <10 mm accounted for 63.2% of the total number of nodules included in our study, resulting in lower diagnostic efficacy than that of the previous studies, as demonstrated by the various study indicators (25). The number of missed malignancies was 1,069 and 1,155, 235 and 273 of which were concomitant LN metastases according to TN-RSS alone in EU-TIRADS and K-TIRADS, respectively. These systems attempt to identify those nodules in which FNA is indicated by considering both TN-RSS and LN-RSS. Accordingly, PMR would decrease from 54.2% to 33.9% in EU-TIRADS and 54.5% to 39.3% in K-TIRADS. Thus, a combination of TN-RSS and LN-RSS may be an effective strategy in the management of thyroid nodules.

As described by 2017 EU-TIRADS and 2021 K-TIRADS, LN-RSS places LNs in one of three risk categories. In each category, LN-RSS provides precise size cutoffs that determine management recommendations (FNA or further action). In our series, malignancy rates in the three risk categories are increasing: normal/probably benign (0% vs. 16.7%), indeterminate (50.5% vs. 44.2%), and suspicious (90.3% vs. 90.3%), according to EU-TIRADS and K-TIRADS. Of note, 19.1% of LNs (150 of 786) did not meet the criteria for any pattern and were thus classified as “not specified” in EU-TIRADS. The malignancy risk of “not specified” was 36.7% (55/150) because of the absence of the hilum, oval shape, and normal size. Thus, 54 of these LNs were located in the central compartment.

According to previous studies, metastatic LNs generally characterized by an increased size, absence of hilum, round shape, abnormal vascularity, and the presence of abnormal internal echogenicity (such as strongly cortical hyperechoic areas, punctate echogenic foci, and cystic changes) (26–28). However, the identification of central cervical metastatic LNs via ultrasonography has encountered significant challenges. There are anatomic areas of the central cervical region that are not well visualized by ultrasonography (2, 29–31). Moreover, central metastatic LNs were always smaller in size than lateral nodes. According to our study, metastatic LNs located in the lateral compartment were more likely to exhibit the previously studied features (cortical hyperechogenecity, cystic changes, punctate echogenic foci, and abnormal vascularity) of thyroid metastatic LNs than those located in the central compartment. In our data, some metastatic LNs in the central neck region were often presented as the presence of the hilum, round shape, and normal size. Although they were both classified as indeterminate categories, they were suggested to be FNA according to EU-TIRADS but failed to meet the size cutoff according to K-TIRADS. Therefore, EU-TIRADS seems to have higher accuracy. EU-TIRADS still has a higher sensitivity without the influence of the “unspecified” LNs. The diagnostic performance of EU-TIRADS was similar to that of K-TIRADS when the “not specified” belonged to the intermediate level. Previous studies revealed that some clinical factors—such as extrathyroidal extension, capsular invasion, and Hashimoto’s thyroiditis—are correlated with cervical metastatic LNs. We expect a model that comprehensively considers clinical factors and US image features. Numerous lymph node metastasis prediction models for thyroid cancer to assess the risks of multiple cervical metastatic LNs patterns have achieved encouraging accuracy. It will help predict the benign and malignant nature of intermediate lymph nodes (32, 33).

Nevertheless, this study had several limitations. First, the assessment of cases was retrospective, and the series included only patients who underwent surgery and FNA at a single tertiary referral center, with selection bias. Second, the percentage of malignant nodules was higher in this study (84.7%) than in other studies. Third, false-negative results of FNA cannot be ignored, as not all patients with benign cytological findings had postoperative pathological confirmation. Fourth, we will endeavor the long-term follow-up to ensure the accuracy of the diagnostics methods tested. Therefore, prospective multicenter studies are needed to verify the generality of our findings.

## 5 Conclusion

Most previous studies have considered only TN-RSS or LN-RSS, which is not logical for daily application. The combination of TN-RSS and LN-RSS is a practical and reasonable diagnostic option. The process meets the original purpose of designing a risk management system for thyroid nodules, which is not to accurately differentiate benign nodules from malignant ones but rather to better manage thyroid nodal diseases. PMR is a simple and effective indicator for assessing the risk stratification system. In this study, the combination of TN-RSS and LN-RSS for the management of thyroid nodules was associated with a reduction in PMR.

## Data availability statement

The original contributions presented in the study are included in the article/supplementary material. Further inquiries can be directed to the corresponding author/s.

## Ethics statement

The studies involving humans were approved by the scientific research and clinical trials ethics committee of The First Affiliated Hospital of Zhengzhou University of China. The studies were conducted in accordance with the local legislation and institutional requirements. The ethics committee/institutional review board waived the requirement of written informed consent for participation from the participants or the participants’ legal guardians/next of kin in accordance with the local legislation and institutional requirements.

## Author contributions

CX: Writing – original draft, Writing – review & editing, Resources. JY: Formal analysis, Writing – review & editing, Methodology, Supervision. CF: Formal analysis, Methodology, Writing – review & editing, Supervision. YC: Data curation, Writing – review & editing. YH: Writing – review & editing, Formal analysis, Methodology. KC: Resources, Supervision, Writing – review & editing.
